# Antibacterial Effect of Triazine in Barrier Membranes with Therapeutic Activity for Guided Bone Regeneration

**DOI:** 10.3390/polym14214482

**Published:** 2022-10-23

**Authors:** Gabriela de Souza Balbinot, Cassiano Mendes Nobre do Espírito Santo, Vicente Castelo Branco Leitune, Fernanda Visioli, Rosane Michele Duarte Soares, Salvatore Sauro, Fabricio Mezzomo Collares

**Affiliations:** 1 Dental Materials Department, School of Dentistry, Universidade Federal do Rio Grande do Sul, Porto Alegre 90035-004, RS, Brazil; 2 Patology Laboratory, School of Dentistry, Universidade Federal do Rio Grande do Sul, Porto Alegre 90035-004, RS, Brazil; 3 Biomaterials Laboratory (PoliBIO), Universidade Federal do Rio Grande do Sul, Porto Alegre 91501-900, RS, Brazil; 4 Group of Dental Biomaterials and Minimally Invasive Dentistry, Department of Dentistry, Cardenal Herrera-CEU Universities, C/Santiago Ramón y Cajal, s/n., Alfara del Patriarca, 46115 Valencia, Spain; 5 Department Interdisciplinary of Bari, Università di Bari “Aldo Moro”, Giulio Cesare Square, 11, 70124 Bari, Italy

**Keywords:** bone regeneration, anti-bacterial agents, triazines, mechanical tests, anti-infective agents

## Abstract

Objective: This study aimed to develop polymer-based barrier membranes based on poly(butylene-adipate-co-terephthalate) (PBAT) with the addition of 1,3,5-triacriloilhexahydro-1,3,5-triazine (TAT). Materials and Methods: Polymeric solutions were used to produce membranes with 5 wt% and 10 wt% of TAT by solvent casting. Membranes without the addition of TAT were used as controls. The membranes were chemically characterized by Fourier transform infrared spectroscopy (FTIR) and thermogravimetry (TGA); surface properties were assessed by profilometry and contact angle; the mechanical behavior was evaluated by a tensile test, and the biological properties were assessed by direct–indirect cell viability and antibacterial activity by *S. mutans* and *S. aureus* colony-forming units. Results: TAT was detected in the FTIR and TGA analyses and modified the top surface of the membranes, increasing their roughness and wetness in both concentrations compared to the control group (*p* < 0.05). The addition of TAT, regardless of concentration, reduced the tensile strength and increased membrane stiffness (*p* < 0.05). The cell viability of 5 wt% TAT and 10 wt% TAT was 86.37% and 82.36%, respectively. All tested concentrations reduced the formation of biofilm on the membranes when compared to the control. Conclusion: The addition of TAT successfully resulted in the antimicrobial ability of PBAT-based barrier membranes, while it maintained acceptable levels of cell viability in membranes with adequate handling and surface properties.

## 1. Introduction

Barrier membranes are essential for the establishment of conditions for guided bone regeneration (GBR) [1,2]. The role of membranes involves the maintenance of space for bone formation by preventing the invagination of soft tissue from the surrounding areas [1]. Although successful clinical outcomes are shown for GBR procedures in bone gain and dental implant success [3,4], postoperative complications are prevalent, and the rate of soft tissue complication is around 17% [5]. Acute infection and abscess are included in these cases and lead to treatment failure, requiring reintervention with the possible need for systemic antibiotic therapy [6].

The currently used commercially available membranes are known to fulfill the requirements for GBR principles but may favor bacterial accumulation due to surface roughness and porosity [6]. The control of membrane properties and the establishment of antibacterial activity for barrier membranes could contribute to effective GBR by preventing or limiting the extent of infection at the surgical site. The use of synthetic polymers allows the production of smooth and non-porous structures with versatile processing, and resorbable polyesters are known candidates for the development of biomaterials [7]. Poly(butylene adipate-co-terephthalate) (PBAT) has been recently proposed for the biomedical field [8,9], showing adequate properties for the application as GBR barrier membranes [10].

The incorporation of antibacterial compounds into the barrier membrane is possible in the manufacturing of PBAT films to produce barrier membranes with therapeutic activity. The compound 1,3,5-trimethylhexahydro-1,3,5-triazine (TAT) was selected in the present study as a known antibacterial compound that has been shown to be effective in materials applied in the oral environment [11,12,13]. Triazines are compounds based on nitrogen heterocycles, and antibacterial activity has been shown for different triazine derivates [14,15], but their effects were not previously studied for barrier membranes and other surgical devices. As cationic antimicrobial peptides, triazines act on the negatively charged bacteria membrane, resulting in its fast disruption with limited resistance with a broad spectrum of activity [16,17], and thus it may hamper bacteria adhesion and biofilm formation in the surgical site, leading to the production of innovative barrier membranes with therapeutic effects. Thus, the antibacterial effect of TAT on PBAT-based membranes was explored in the present study. The developed materials were formulated and evaluated for their physicochemical and biological properties.

## 2. Materials and Methods

### 2.1. Barrier Membrane Production

**Figure 1:**
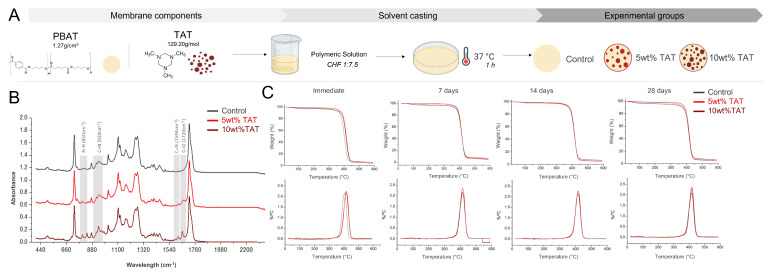
(**A**) Schematics of membrane fabrication via solvent casting. Chemical characterization of developed materials by (**B**) FTIR analysis with the characteristic bands in TAT and (**C**) the thermal behavior of experimental barrier membranes before and after immersion in SBF.

Barrier membranes were produced by solvent casting. Poly(butylene adipate-co-terephthalate) (PBAT—1.27 g/cm^3^ at 23 °C density, Ecoflex^®^ F Blend C1200; BASF Corporation, Florham Park, NJ, USA) pellets were mixed in chloroform at 1:7.5 (*v*/*w*) for 24 h. Triazine (1,3,5-trimethylhexahydro-1,3,5-triazine (TAT), Merck KGaA, Darmstadt, Germany) was added to the polymeric solution at 5 wt% and 10 wt% concentrations. Casting took place in glass slides, and the solvent was allowed to evaporate for 1 h (Figure 1A). Membranes without the addition of TAT were used as control.

### 2.2. Chemical Characterization

Materials were evaluated through Fourier-transform infrared spectroscopy (FTIR) to assess the chemical composition of developed membranes. A spectrometer (Vertex 70-Bruker Optics, Ettlingen, Germany) was used with an attenuated total reflectance device (Platinum ATR-QL; Bruker Optics). Membranes (4 mm diameter and 1 mm height; n = 1) were placed in close contact with the ATR device, and the analysis was performed in the 400–4000 cm^−1^ range with 16 scans for each sample. The thermal behavior of materials was assessed via thermogravimetric analysis (TGA-Discovery, TA Instruments, New Castle, DE, USA). Samples were heated up to 600 °C at a rate of 10 °C min^−1^ under nitrogen purge (25 mL·min^−1^). The weight loss % (TGA) and the differential thermogravimetric (DTG) were calculated in samples immediately after preparation and after 7, 14, and 21 days of immersion in simulated body fluid (SBF), prepared according to standard protocol [18].

### 2.3. Mechanical Behavior

A tensile test was performed with hourglass specimens that were prepared according to ASTM D638-02 type IV plastics [19]. Samples were tested in a mechanical test machine (Shimadzu EZ-SX, Shimadzu Corp., Kyoto, Japan) at a 1 mm/min crosshead speed immediately after preparation and after a 28-day immersion in an SBF solution [18]. The strain–stress behavior was used to calculate the ultimate tensile strength, the Young’s modulus, and the elongation rate of developed membranes.

### 2.4. Surface Properties

The sessile drop method was used to measure the water contact angle on the samples’ top and bottom surfaces. A 20 µL distilled water drop was poured into the samples (6 mm diameter × 0.2 mm height; n = 3) in an optical tensiometer (Theta Line, Biolin Scientific, Stockholm, Sweden). A high-resolution camera monitored the water’s behavior on the material surface, and measurements of the contact angle were performed after 10 s by image software (OneAttension, Biolin Scientific, Stockholm, Sweden), where the angle between the drop and the sample was calculated for the left and right side of the drop. The average contact angle in each drop was used, and three measurements were performed per sample. The surface roughness was measured with optical profilometry (Optical Profiler ContourGT, Bruker). The samples (6 mm diameter × 0.2 mm height; n = 3) were scanned with 5× monochromatic light by vertical scanning interferometry on both membrane sides in a standardized area (1260 µm × 1260 µm). The Ra parameter was measured as the samples’ arithmetic average of the surface roughness profile.

### 2.5. Biological Properties

Cell cultures were performed with the preosteoblastic MC3T3-E1 cell line (Banco de Células do Rio de Janeiro, Rio de Janeiro, Brazil). The MC3T3-E1 cells were cultivated with α-MEM supplemented with 10% fetal bovine serum and 1% penicillin (Thermo Fischer Scientific, Waltham, MA, USA) at 37 °C at 5% CO_2_. No additional supplementation was added to the media, as recommended for this cell line. Cell proliferation was assessed by the sulphorodhamine B (SRB) assay with a direct–indirect method. Material extracts were produced with membrane specimens (6 mm diameter × 0.2 mm height; n = 3) immersed in a culture medium for 24 h. Cells were cultivated at 5 × 10^3^ density in a 96-well plate, and treatments were performed for 72 h. Cells were fixed and stained with 0.4% SRB solution. The SRB dye was quantified at 560 nm in a microplate spectrophotometer (Multiskan GO, Thermo Fisher Scientific, Waltham, MA, USA). The results in wells treated with extracts were normalized for the absorbance in wells cultivated for the same time without material immersion.

Antibacterial ability was evaluated against *Streptococcus mutans* (NCTC 10449) and *Staphylococcus aureus* (ATCC 25923). An *S. mutans* suspension was used after 18 h incubation in brain heart infusion (BHI) broth, while an *S. aureus* suspension was used after 24 h in BHI. Six specimens (4 mm diameter × 1 mm thickness) were immersed in each suspension in 48-well plates and incubated at 37 °C, and biofilms were allowed to grow for 24 h. For biofilm quantification, three specimens were vortexed for 1 min in a micro-tube containing 900 μL of saline solution, and dilutions were made up to 10^−6^ before two 25 μL-drops of each dilution were platted in BHI agar Petri dishes. Bacteria growth at 37 °C took place for 48 h for *S. mutans* and 24 h for *S. aureus*. For planktonic analysis, the inocula were collected, dilutions were made up to 10^−6^, and bacteria were platted as described for the biofilm analysis. In this case, a negative control, without material immersion, was used to assess the bacteria suspension. The number of colony-forming units (CFU) was visually counted by optical microscopy and transformed to log10 CFU/mL.

### 2.6. Statistical Analysis

FTIR, TGA, DTG, and representative images of RA were descriptively analyzed. Data were submitted to the normality test by Shapiro–Wilk. One-way ANOVA was used as a parametric method to assess the difference between groups in the biological properties assessment. Two-way ANOVA was used to analyze mechanical behavior and surface properties data. Tukey was used as a post hoc test in all analyses. All analyses were conducted at 95% significance.

## 3. Results

The chemical characterization is shown in Figure 1. FTIR data showed the characteristic N–H (850 cm^−1^), C=N (920 cm^−1^), C=N (1594 cm^−1^), and C=O (1720 cm^−1^) that are assigned to vibrations of nitrogen bonding on the triazine ring and the ester group in the methacrylate terminals. TGA and DTG (Figure 1C) showed the main weight loss in PBAT (~370 °C–450 °C) with a minor loss of 0.95% and 3% between 60 and 150 °C in membranes with 5 wt% and 10 wt% TAT, respectively.

**Figure 2:**
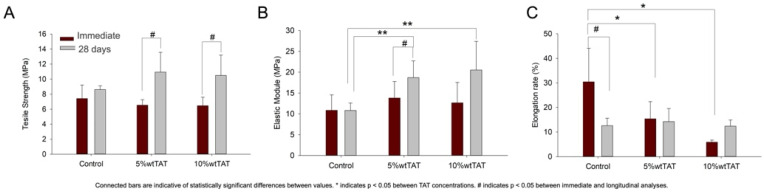
Mechanical behavior of developed barrier membranes. The addition of TAT modified the tensile strength (**A**), the elastic modulus (**B**), and the elongation rate (**C**) in PBAT films. Bars are a description of average values, while error bars represent standard deviations. Connected bars are indicative of statistically significant differences between values. * Indicates statistically significant differences between TAT concentrations and # between immediate and longitudinal analyses. * *p* < 0.05; ** *p* < 0.01.

The mechanical behavior of developed materials was presented as the ultimate tensile strength (Figure 2A), elastic modulus (Figure 2B), and elongation rate (Figure 2C). The 28 days of immersion in SBF increased the tensile strength and elastic modulus of developed materials in the TAT-containing membranes (*p* < 0.05). The elongation rate was decreased after immersion for the control group with comparable values for the membranes with TAT addition.

**Figure 3:**
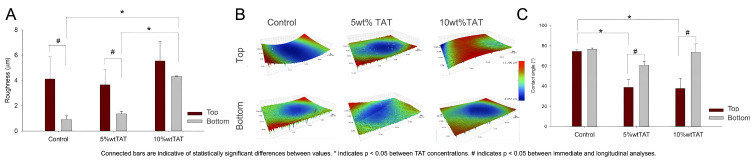
Surface properties on the top and bottom of developed membranes. (**A**) Average values for surface roughness (RA) via profilometry with representative images of the analyzed area showing the distribution of roughness values for higher values (green to red) and lower values (green to blue) in a range of −8.5μm to 12.8μm (**B**). Water contact angle in the developed materials (**C**). Bars are a description of average values, while error bars represent standard deviations. Connected bars are indicative of statistically significant differences between values. * Indicates statistically significant differences between TAT concentrations and # between immediate and longitudinal analyses.

No statistically significant difference was observed in the RA values on top of membranes, while the addition of 10 wt% TAT modified the bottom surface in the profilometry analysis, as observed in Figure 3A,B. The water contact angle was reduced in the membrane top surface regardless of the concentration, ranging from 74.27° in the control group to 37.62° in the 10 wt% TAT group.

**Figure 4:**
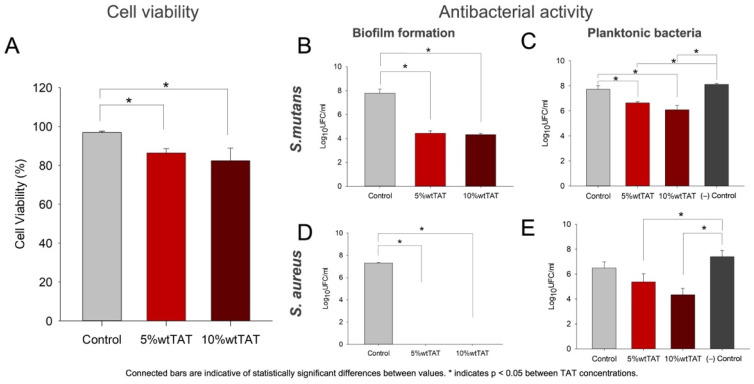
Cell behavior and antibacterial activity analysis. (**A**) Cell viability in MC3T3-E1 cells after 72 h of culture via SRB analysis. Antibacterial activity of experimental barrier membranes against *S. mutans* (**B**,**C**) and *S. aureus* (**D**,**E**) in log10 CFU/mL. Bars are a description of average values, while error bars represent standard deviations. Connected bars are indicative of statistically significant differences between values. * Indicates statistically significant differences between TAT concentrations and # between immediate and longitudinal analyses.

A reduction in MC3T3-E1 viability was observed in groups with TAT (Figure 4A; *p* < 0.05). The cell viability of 5 wt% TAT and 10 wt% TAT was 86.37% and 82.36%, respectively. The antibacterial activity was observed for both *S. mutans* and *S. aureus* cultures, mostly when the biofilm formation was assessed (Figure 4B,D). A 3 log10 UFC/mL reduction was observed in the *S. mutans* biofilm formation in both 5 wt% and 10 wt% TAT when compared to the control (Figure 4B), while no colony formation was detected on the *S. aureus* culture with membranes containing TAT (Figure 4D). A statistically significant reduction in CFU/mL was observed in *S. mutans* planktonic bacteria in membranes with TAT when compared to the control group and negative control.

Within these results, it is possible to observe that the newly developed membranes were able to control the biofilm formation when two bacteria strains were tested, maintaining the biocompatibility and physicochemical properties of PBAT-based films to produce antibacterial barrier membranes.

## 4. Discussion

Barrier membranes are studied as a possible vehicle for the local delivery of antibacterial compounds in the development of GBR materials with therapeutic activity to control post-operative complications [20]. In this study, 1,3,5-triacriloilhexahydro-1,3,5-triazine (TAT) was incorporated into poly(butylene adipate-co-terephthalate) (PBAT) membranes, and the resultant materials presented antibacterial activity with controlled physicochemical properties for their application in GBR procedures.

Solvent casting has been a popular manufacturing technique in polymer processing [21], and its application for PBAT membranes has been previously described [10]. The addition of TAT was observed in the chemical characterization by FTIR (Figure 1B), mostly related to the presence of nitrogen bonding in the triazine rings [15,22], confirming the incorporation of antibacterial compounds into the PBAT structure, without compromising the PBAT characteristic bonding structure [23]. FTIR data also showed that the production methods successfully removed the solvent used for the polymer, and that it did not modify the structure of the TAT incorporated into the polymeric structure. The thermal behavior of developed membranes was shown in Figure 1C and corroborated the FTIR results, as it showed a characteristic behavior for weight loss in PBAT [24] along with known degradation that was observed in TAT. The weight loss in PBAT was related to its degradability, known in several applications [25,26], and it was not modified in the 28-day analysis in this study. TAT also presented little changes in degradation over time (Figure 1C). The loss of mass, in this case, may indicate possible degradation of TAT, although this is not known for this monomer composition, and further analysis may elucidate the degradation behavior of TAT into resorbable compounds. While no chemical interaction between TAT and PBAT was observed, the antibacterial monomer is likely to be entrapped into the polymer network, as evidenced by the little modification observed in TGA analysis after immersion, as well as by the antibiofilm properties observed for different bacteria species (Figure 4B,D).

The maintenance of the chemical properties of PBAT was also observed in the mechanical behavior of developed membranes (Figure 2). PBAT is known as a flexible polymer due to its unique structure comprising adipate and terephthalate units and the control in the crystallization of these units [8,27]. The addition of TAT maintained the strength and stiffness in developed membranes in an immediate analysis (Figure 2A,B), and over time an increase in stiffness was observed for the 10 wt% TAT group, which corroborated the maintenance of the PBAT chain structure observed in FTIR, while the modifications over time could be assigned to TAT degradation, as shown in TGA (Figure 1). The flexibility could contribute to the application of these materials as barrier membranes in GBR. The handling and adaptation of membranes depend on the ability of materials to be adapted in the surgical site, fitting it to the bone defect for tissue regeneration. Moreover, the membrane has a major role in the occlusion of the defect and space maintenance for bone growth [28,29], and controlling the strength of these materials may contribute to the establishment of the core principles of GBR [1,2].

The adaptation of membranes in the surgical site must also consider the wetting in the developed membranes as an essential aspect to allow the interaction of membranes with the surrounding tissues. The presence of TAT also modified the surface properties both in the top and bottom of membranes (Figure 3). The RA values observed on the top and bottom surfaces are related to the manufacturing by solvent casting. The bottom surface is in contact with a glass slide, and thus the polymer is expected to present a smoother surface, while the roughness obtained from the addition of TAT is found on the top of membranes, as observed in RA average values (Figure 3A) and representative profilometry images (Figure 3B). The wetting of samples was also modified by TAT addition and the analyzed surface (Figure 3C). While the top and bottom surfaces of the control group presented contact angle values that were suitable for PBAT films [10,30], the presence of TAT reduced the contact angle. Although an increased interaction is desired when membranes are in contact with bone tissue, for a good intercommunication with cells and coagulum into the defect, the need for a barrier effect may be favored by lower wettability in contact with the soft tissues. The key to successful GBR is to avoid the penetration of soft tissue from surrounding areas to the bone defect, protecting bone growth [28,31].

The biological behavior of developed materials was assessed by their influence on cell and bacteria viability, as shown in Figure 4. While PBAT has been recently proposed for biomedical applications, it is known that no cytotoxic effect is found in vitro [9,10,25], with no adverse effects found in vivo [9,32]. In this study, the PBAT also did not reduce the cell viability of a preosteoblasts cell lineage, while a statistically significant reduction in cell count was found for TAT-containing groups (*p* < 0.05). Cells were cultivated in complete α-MEM culture media without osteogenic differentiation to maintain the proliferation potential in the in vitro cell culture assay. While antibacterial compounds may be related to cytotoxic effects [33], all groups reached >80% values in the SRB analysis, which is suitable for materials designed for biological applications [34,35,36].

The antibacterial effect, known for triazine compounds in different applications [11,12,13,15,17,22,37], was observed in this study for *Streptococcus mutans*, which are among the most prevalent bacterial in the oral environment [38,39] and was selected in this study to confirm the ability of these materials to reduce the colony formation of streptococci species [39]. *Staphylococcus aureus* cultures were studied as prevalent species in infected sites in epithelial tissues [40], including the oral mucosa [41], being thus related to post-operative complications that are related to infections in GBR procedures [5]. For both *S. mutans* and *S. aureus*, we observed a reduction >3 log_10_ CFU/mL in the biofilm analysis, which shows that the addition of TAT bactericidal effects may be a tool to control the bacteria adhesion and consequently the biofilm formation on membranes surfaces. The antibiofilm capacities of developed membranes are related to the entrapment of TAT into the PBAT structure and explain the slight antibacterial effect observed for the planktonic bacteria, shown in Figure 4B,D. This effect shows that antibacterial compounds act on the material surface, probably not being released on media during the analysis, and thus maintenance of the effect could be expected in this short-term analysis.

Controlling the bacteria adhesion and biofilm formation on the membrane surface may be a strategy for the local control of infection in GBR procedures [28]. Antibacterial barrier membranes could contribute to the maintenance of defect closure during the regenerative process, avoiding reinterventions due to post-operative complications related to infections and reducing the need for systemic antibiotic therapy. More than guaranteeing the principles of GBR, the potential control of infections after surgical procedures may contribute to better cost-effectiveness of treatments and control of antimicrobial resistance. The successful production of a TAT-containing PBAT-based barrier membrane in this study could contribute to the development of materials with therapeutic activity with a broad spectrum of antibacterial effects and controlled physicochemical properties.

## 5. Conclusions

TAT is a candidate to produce barrier membranes with effective control in bacteria accumulation, and the combination of this compound with PBAT allowed the production of antibacterial materials with controlled properties for a barrier membrane effect.

## Data Availability

Data presented in this study are available on request from the corresponding author.
